# Personal health and nutrition information-seeking attitudes and behaviours of first year Canadian and United States veterinary students

**DOI:** 10.18849/ve.v7i2.543

**Published:** 2022-04-13

**Authors:** Shelby A. Nielson+, May K. Kamleh+, Peter D. Conlon+, Jennifer E. McWhirter+, Elizabeth A. Stone+, Deep K. Khosa+

**Keywords:** DIETARY HABITS, HEALTH INFORMATION, HEALTH INFORMATION-SEEKING BEHAVIOURS, INFORMATION SEEKING, STUDENT NUTRITION, STUDENT WELL-BEING, VETERINARY STUDENTS

## Abstract

**Objective:**

To identify the primary sources of information first year Canadian and US veterinary students relied on for their personal health and nutrition information, and to explore their attitudes towards, and perceptions of, health information resources.

**Background:**

Though the animal health information-seeking behaviours (HISB) of veterinary students have been explored, research regarding personal HISB of this professional student population is limited.

**Evidentiary value:**

Participants were first year veterinary students (n=322) at the five Canadian veterinary schools and five randomly selected US veterinary schools. An online questionnaire was used to gather students’ demographic information, sources of health and nutrition information, and information-seeking attitudes and perceptions. This study may impact practice at the institutional level for veterinary educators.

**Methods:**

STATA 15.1^©^ was used for quantitative analysis; involving multivariate logistic regression models, univariate analyses, and measures of frequency.

**Results:**

Results indicated high reliance on the Internet for personal health 213/322 (66%) and nutrition 196/322 (61%) information. While respondents revealed high trust levels in dietary recommendations from family doctors, 132/322 (41%) of students revealed their doctor did not provide any information on healthy diets. Students who reported the use of peer-reviewed journal articles for personal nutrition information were at greater odds of having confidence in knowing where to find nutrition information (Odds Ratio [OR] = 6.61, p<0.001).

**Conclusion:**

Participating students reported a high reliance on the Internet search engine Google, and a general lack of guidance from medical professionals regarding general health needs.

**Application:**

Veterinary schools should consider this information to enhance student information literacy skills, particularly to facilitate personal HISB, and consequently help in management of personal health throughout the growing demands of the programme.

## INTRODUCTION

Modern technological advances have provided wide access to health and nutrition information for the general population. This may be especially true for university students who have frequent access to the Internet and rely on it throughout their education. Research suggests that the use of the Internet may influence health information-seeking behaviours (HISB) (Chen et al., 2018), which is recognised as the process of gathering information about health (Mukherjee & Bawden, 2012). Such information-seeking behaviours have been shown to inform decision-making and encounters with health professionals as well as personal health behaviours (Chen et al., 2018; and McKinley & Wright, 2014). Given its fundamental role and influence on overall health and well-being, nutrition is an important component of HISB (Lichtenstein et al., 2006). Understanding where and how individuals seek health-related information can subsequently advise targeted initiatives to promote individual and population health (Mukherjee & Bawden, 2012).

The HISB of post-secondary students (those enrolled in education beyond high school) have been somewhat explored, and results have demonstrated a trend towards greater reliance on the Internet for health information over time. A 2005 study noted that less than half of college students reported they had frequently searched the Internet for health information (Escoffery et al., 2005). Although in a 2011 study, Percheski & Hargittai found that 831/1060 (78%) of first year undergraduate students indicated the Internet as a health information resource, more traditional health information resources such as family and friends and health professionals remained highly ranked. More recently, results by Basch et al. (2018) indicated that 191/258 (74%) of college students were most likely to often or always use the Internet for health information, and reported more reliance on this source than on medical professionals and family and friends. These results were consistent with those of Rennis et al. (2015), who found that the Internet was the most consulted health information source by college students. Further, research focusing on students in advanced healthcare programmes such as medicine and dentistry have also revealed a high use of the Internet to support their learning (O’Carroll et al., 2015; and Schleyer et al., 2012).

Though some researchers have examined the HISB of veterinary students specifically, most existing literature has focused on the information search habits in regard to animal health information. Results of those studies have similarly identified a reliance on the Internet (Dale et al., 2011; Weiner et al., 2011; and Lai et al., 2019). While online sources may be a convenient way for students to access information related to animal health, students may also access unreliable information, thereby potentially affecting student learning. Previous research has identified inconsistencies in the accuracy and completeness of online animal health information, including focused topic areas such as canine cruciate ligament disease, canine osteoarthritis, and canine anaesthesia (Taggart et al., 2010; Jehn et al., 2003; and Hofmeister et al., 2008). Similar trends of unreliability have been identified in online human health information, where authors concluded that websites provided incomplete or inaccurate information despite the overall quality of such websites ranking as fair (Reynolds et al., 2018). Given that research studying first semester veterinary students revealed that students used few criteria to evaluate the quality of websites and provided information prior to use (Weiner et al., 2011), it is important to explore what resources veterinary students are using to learn about their own health and nutrition. In a highly demanding programme such as veterinary medicine, it is critical for students to make beneficial nutrition decisions and stay healthy to best support themselves as the requirements of the programme intensify. Presently, however, little is known about how and why veterinary students make health and nutrition decisions. This study aims to help address gaps in this knowledge. As an exploratory study, the objectives were to:

1. identify the sources used by first year veterinary students to learn about their health and nutrition; and
2. explore students’ attitudes and perceptions of health and nutrition information sources during their first year of veterinary school.

Outcomes from this study may inform future research and intervention strategies to promote veterinary student information literacy skills, including personal HISB, upon entering the programme in order to make progressive decisions to best support personal health and well-being, and accomplish educational and professional goals.

## METHODS

Study design

Students commencing their first year of veterinary medicine studies (DVM or its equivalent) at the five Canadian veterinary schools and five randomly selected US veterinary schools were recruited in this multi-centre cross-sectional study. They were invited to complete an online questionnaire between September 2016 and November 2016. Since the veterinary schools were randomly selected, the authors of this paper have chosen to omit the names of the individual veterinary schools. This study received University of Guelph’s Research Ethics Board approval (REB # 16JA039), and ethics approval from all participating schools.

Questionnaire Design

A review of the existing literature and focus group discussions with pre-veterinary (undergraduate students with interest in attending veterinary school) and veterinary students (n=84) were used to develop a questionnaire. It included questions about demographics, sources of health and nutrition information, and health and nutrition information-seeking attitudes and perceptions. For the purposes of this study, health information was defined as the seeking of information regarding health status, health-promotive activities, or social or personal sense of health overall. Nutrition information for the purposes of this study referred to all aspects of nutrition-related health inquiries. Questions were in multiple-choice or 5-point Likert scale formats. In the case of multiple-choice questions, respondents had the opportunity to select more than one response and further specify their response using an ‘other’ category. The survey questions were distributed to participants as part of a larger research study (Kamleh et al., 2020b).

Demographics

Using multiple-choice questions, first year veterinary students were asked their age, gender, previous programme of study, if they were a domestic or international student, and their future career plans.

Sources of health and nutrition information

Using multiple-choice questions, respondents were asked to identify primary information sources relied on for both their personal health, as well as their personal nutrition needs (e.g. personal healthcare provider, books / magazines, the search engine Google, family member). Google was the only Internet search engine offered in these response options.

Health and nutrition information-seeking attitudes and perceptions

Respondents were asked to use 5-point Likert scales to rate how much knowledge they believed to exist on human nutrition (1 = *none*, 5 = *very high amount*) and their confidence in knowing where to find information about their own nutrition (1 = *very difficult*, 5 = *very easy*). Students were additionally asked to rate their levels of trust in various nutrition information sources (e.g. messages the media portrays with regards to nutrition, online sources for nutrition, family doctors recommending certain diets) using a 5-point Likert scale (1 = *none at all*, 5 = *a great deal*). Finally, respondents’ views on the quality / quantity of information provided to them about healthy eating by their doctor was explored using a 5-point Likert scale (1 = *My doctor does not provide information about healthy diets*, 5 = *Information is more than I need*).

Questionnaire distribution

The survey was initially piloted in English at the home research department with six veterinary students during the second semester of their programme. The feedback received from pilot testing was used to further refine the survey questions and establish content validity. Internal consistency of the questionnaire instrument was satisfactory (Cronbach’s alpha = 0.76). A translator was used to develop a French version of the survey. The final questionnaire was uniformly distributed via Qualtrics^©^ (Provo, Utah) by email invitation from each school’s student affairs office. A cover letter was included within the questionnaire to ensure that uniform information was provided to all participants, including a statement that described the general risks of managing data over Internet transit. Students were required to read the cover letter and provide consent to participate in the survey. The questionnaire was provided in English to all the schools and additionally in French to the French-language Canadian veterinary school. The survey was anonymous, and students were provided with an external link to enter a separate, optional prize draw upon submission of the questionnaire.

Questionnaire data analysis

Questionnaire data were numerically coded and entered into an Excel spreadsheet for quantitative analysis using STATA 15.1^©^ (2017, College Station, Texas). The initial univariate analysis involved verifying distributions. The decision to dichotomise variables that were measured on a 5-point Likert scale was made prior to analysis to support interpretability of results. For ‘the amount of information to exist on human nutrition’, categories 1, 2 and 3 (*none, small amount, unsure*) were combined, as were categories 4 and 5 (*moderate amount, very high amount*) to create a dichotomous variable (0 = *small amount*, 1 = *large amount*). ‘Confidence in finding personal nutrition information’ was dichotomised, where categories 1, 2 and 3 (*strongly unconfident, moderately unconfident, neither confident nor unconfident*) were combined, as were categories 4 and 5 (*moderately confident, strongly confident*) to create a dichotomous variable (0 = *unconfident*, 1 = *confident*). ‘Trust in different nutrition information sources’ was dichotomised, so that categories 1, 2, and 3 (*none at all, a little, a moderate amount*) and categories 4 and 5 (*a lot, a great deal*) were combined to create a dichotomous variable (0 = *not trusted*, 1 = *trusted*). Categories 1, 2 and 3 (*my doctor does not provide information about healthy diets, information and advice is insufficient, information is neither excessive nor insufficient*) for the question about the ‘quality / quantity of information provided about healthy diets by a family doctor’ were combined, as were categories 4 and 5 (*information and advice is sufficient, information is more than I need*) were combined to create a dichotomous variable (0 = *information is insufficient*, 1 = *information is sufficient*). The variable ‘age’ was reduced from six to three categories where categories 1 and 2 (<20 and *20–22*), 3 and 4 (*23–25* and *25–27 *[*sic*]), and 5 and 6 (*28–30* and >30) were combined to create three overall categories (1 = *≤ 22*, 2 = *23–27*, 3 = *≥ 28*).

Predictor variables were pre-screened using a liberal p-value (£ 0.2) for inclusion in the respective model. To screen for any pairwise correlations between the independent categorical variables, a Spearman’s correlation analysis was performed. A likelihood ratio test was completed prior to removing any categorical variables that did not yield statistical significance (p-value ≤ 0.05) for inclusion in the respective final multivariable logistic regression model. Univariate analyses were used to examine the effect of different nutrition sources on perceived amount of information to exist on human nutrition, and two logistic regression models were constructed to evaluate the effect of predictor variables on confidence in nutrition information-seeking abilities, and trust in family doctors recommending certain diets.

Measures of frequency were evaluated for: primary sources of nutrition information; primary sources of general health information; amount of information students think exist on human nutrition; perceived confidence in knowing where to find personal nutrition information; perceived difficulty in finding personal nutrition information; amount of trust placed in different nutrition information sources; and perceived quality / quantity of information regarding healthy diets received from family doctor. The non-parametric Mann-Whitney Rank Sum test was performed to check for significant differences between information sources used for personal nutrition and health information. Significance was considered at p ≤ 0.05.

## RESULTS

Response rate and demographics

Of the 942 students who received the questionnaire, 322 completed it yielding a 34% response rate. Most (150/322 [47%]) of the respondents were ≤22 years old, with 45% (145/322) between 23–27 and only 8% (27/322) were ≥*28*. The majority (285/322 [89%]) of students identified as female, with only 11% of respondents identifying as male (37/322). Canadian respondents were slightly more represented (160/322, 50%) than American respondents (156/322, 48%), with the remaining students either identifying as an International Resident (4/322 [1%]) or preferring not to declare their area of residence (2/322 [1%]). Animal Biology (142/322 [44%]) was the most common previous programme of study. The majority of respondents planned to pursue a career in a companion animal exclusive practice (105/322 [33%]) or rural community practice (82/322 [25%]) (Table 1).

**Table 1:**
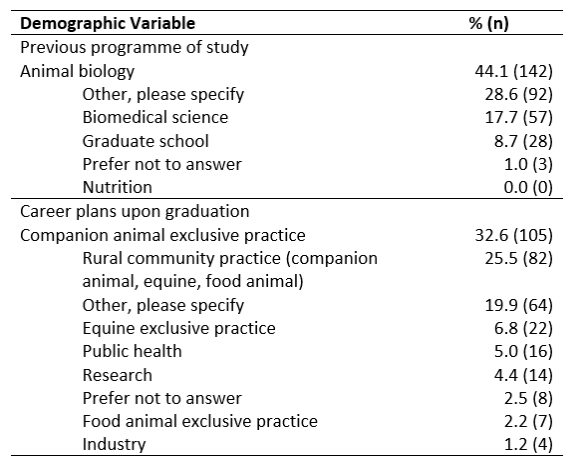
Previous programme of study and career plans upon graduation of first year Canadian and US veterinary students who completed questionnaire on nutrition attitudes and behaviours (n-=322).

Sources of health and nutrition information

**Figure 1:**
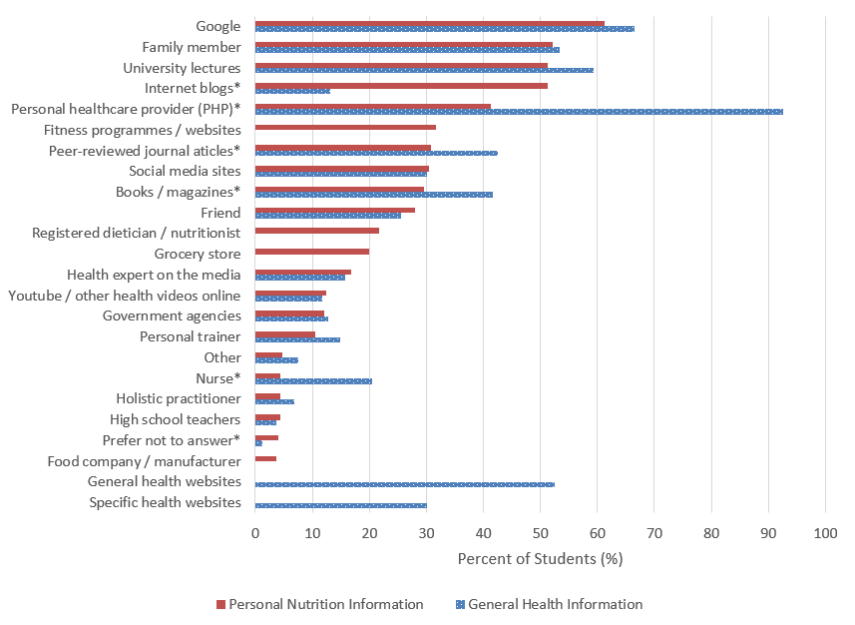
Primary sources of information utilised for general health and personal nutrition needs by first year Canadian and US veterinary students (n=322) retrieved from questionnaire that assessed health attitudes and behaviours. Students could slect more than one response option from these questions (*p<0.05, derived from Mann-Whitney Rank Sun test).

As shown in Figure 1, 93% (298/322) of students reported the use of a personal healthcare provider (PHP) as a primary information source for their general health, but only 41% (133/322) of students relied on their PHP as a primary resource for their personal nutrition information. More than half of respondents reported using the Internet search engine Google as a primary information source for their personal health (214/322 [66%]) and personal nutrition (197/322 [61%]) needs. Family members and university lectures were also reported as primary information sources by over 50% of students, with family members relied upon by 53% (172/322) for general health information and 52% (168/322) for nutrition information, and university lectures relied upon by 59% (191/322) and 51% (165/322) for general health and nutrition information, respectively. There were significant differences in the reported use of books / magazines, Internet blogs, nurses, peer-reviewed journal articles, and PHPs for personal health versus nutrition needs. Additionally, there was a significant difference in students who preferred not to answer with regards to their primary information sources for personal health versus nutrition needs (p <0.05). A complete comparison of information sources utilised by first year veterinary students for their general health and nutrition is shown in Figure 1.

Previous programme of study and career plans upon graduation of first year Canadian and US veterinary students who completed questionnaire on nutrition attitudes and behaviours (n-=322).

**Table 2:**
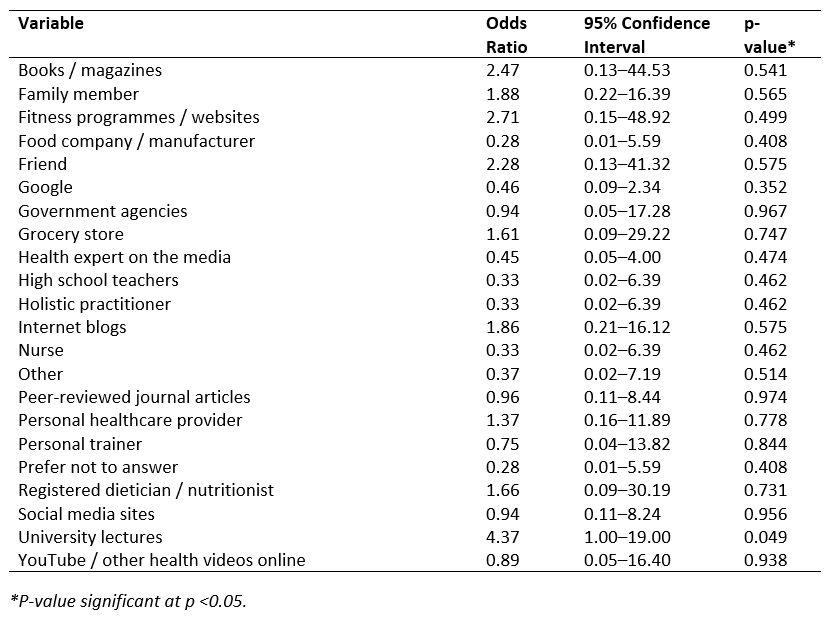
Univariate analyses results of primary nutrition information sources influencing first year Canadian and US veterinary students' self-reported perceived amount of information to exist on human nutrition showing variables, odds ratio, 95% confidence intervals and significance values (n=322)

Students were asked how easy it is to find information about their own nutrition. The majority (138/322 [43%]) of respondents thought this task to be moderately easy, with 16% (50/322) believing it to be very easy. 11% (37/322) of students thought finding information about their own nutrition to be moderately difficult, and very few (5/322 [2%]) thought it to be very difficult. One quarter (79/322) of respondents were unsure how easy they perceived finding information about their own nutrition to be.

Using a series of subscale question statements, students were asked to rate their level of trust with respect to regulatory standards, dietary recommendations by health professionals, messages portrayed by the media, and online information sources (Table 3). Advice from a registered dietician for personal nutrition ranked relatively high in trust by respondents, with most trusting them either ‘a lot’ (116/322 [36%]) or ‘a great deal’ (122/322 [38%]). Messages that the media portrays and advice from a health professional who is sponsored by a certain food company were only trusted ‘a little’ by almost half of respondents (135/322 [42%]). Students indicated a general trust towards diet recommendations from family doctors, with 34% (108/322) and 33% (106/322) trusting this information ‘a lot’ or ‘a moderate amount’, respectfully. The age of first year veterinary students was significantly associated with having trust in family doctors recommending certain diets (Table 4). Younger students were more likely to trust this information, with those aged *≤ *22 three times as likely (p <0.02), to trust dietary recommendations from family doctors than those aged ³ 28. There was no significant difference in trust between respondents aged *≤ *22 and 23–27. Students who indicated their PHP as a primary source for their own nutrition information were also more likely to trust family doctors recommending certain diets (OR = 2.02, p <0.03).

**Table 3:**
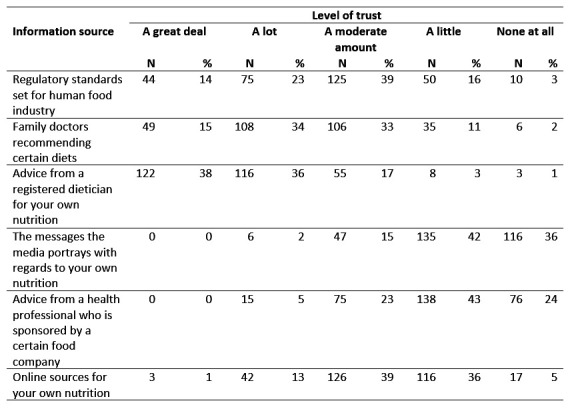
Amount of trust placed in different information sources as self-reported by first year Canadian and US veterinary students as part of questionnaire on health and nutrition attitudes and behaviours (n=322).

**Figure 4:**
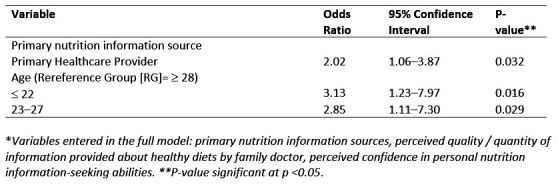
Reduced* multivariable logistic regression results of variable influencing first year Canadian and US veterinary students' self-reported trust in family doctors recommending certain diets showing significant variables, odd ratios, 95% confidence intervals, and significance values (n=322).

**Figure 2:**
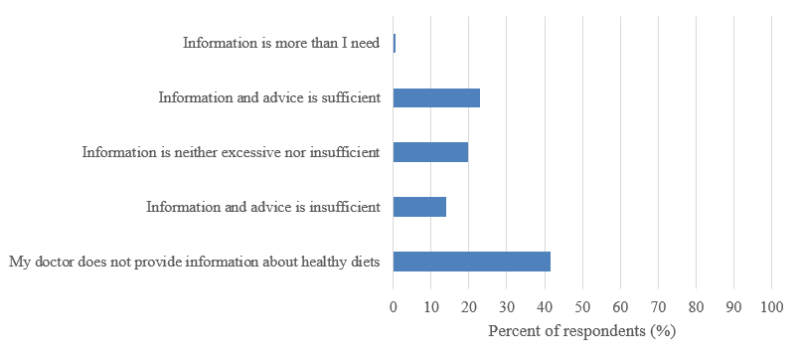
First year Canadian and US veterinary students' response to the question, 'What is your view on the quality / quantity of information provided about healthy diets by your family doctor?' on a questionnaire that assessed health and nutrition attitudes and behaviours (n=322).

When asked about the quality and quantity of information regarding healthy diets first year students received from their family doctor, almost half of respondents (134/322 [42%]) indicated that their doctor did not provide any information about healthy diets. Figure 2 presents a complete breakdown of students’ views on the nutrition information provided to them by their family doctor.

Almost half (157/322 [49%]) of respondents acknowledged that they were moderately confident in knowing where to find information relating to their nutrition, with 18% (57/322) of respondents being strongly confident. 19% (62/322) of students were neither confident nor unconfident in knowing where to find information about their own nutrition, 8% (27/322) were moderately unconfident, and only 2% (6/322) of students indicated that they were strongly unconfident in this ability. Results of significant variables associated with students’ confidence in their nutrition information-seeking abilities is presented in Table 5. Of note, reliance on peer-reviewed journal articles as a primary information source significantly influenced students’ confidence in their own nutrition information-seeking abilities (OR = 6.61, p <0.001). Students who perceive a large amount of information to exist on human nutrition were also more likely to have confidence in their information-seeking abilities with regards to their own nutrition (OR = 5.25, p <0.001).

**Table 5:**
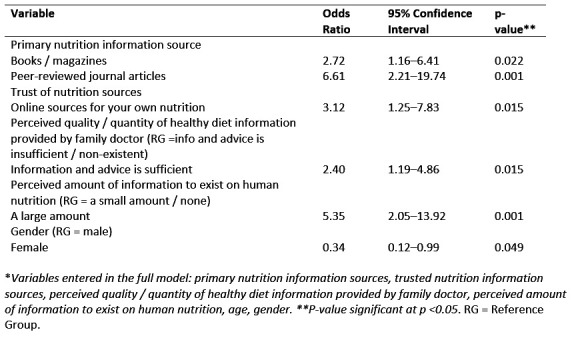
Reduced* multivariable logistic regression results of variables influencing first year Canadian and US veterinary students' self-reported confidence in personal nutrition information-seeking abilities showing significant variables, odds ratios, 95% confidence intervals, and significance values (n=322).

## DISCUSSION

Sources of health and nutrition information

Findings of this study offer an understanding of first year veterinary students’ attitudes and behaviours regarding their personal health and nutrition information-seeking habits. Though PHPs were the most commonly reported primary information source by students for general health information, the Internet search engine Google was the next-most reported source and was the highest-ranked primary information source for students’ personal nutrition needs. Research indicates that young, highly educated, healthy females may be especially active in online HISB in the US population, and these predictors have remained consistent over time (Percheski & Hargittai, 2011; Rice, 2006; and Song & Oh, 2015). These demographic identities align well with our study population, with a distinct female gender bias (285/322 [89%]). This may be one reason why online information sources were highlighted in our findings and reflects the gender composition at other veterinary colleges (Royal et al., 2018). Moreover, high reliance on the Internet is consistent with existing literature investigating health and nutrition information-seeking behaviours amongst diverse student populations (Basch et al., 2018; O’Carroll et al., 2015; and Schleyer et al., 2012), and in the animal health information-seeking habits of veterinary students (Weiner et al., 2011; Lai et al., 2019; and Kamleh et al., 2021).

Information-seeking attitudes and perceptions

The Internet may be an advantageous resource for students seeking health information by being easily accessible, discrete, and acting as a host to various communities to facilitate supportive interactions (Rennis et al., 2015; Giles & Newbold, 2013; Ho et al., 2014; and Lagoe & Atkin, 2015). Despite such benefits, use of Internet search engines, such as Google, can produce an overwhelming amount of results which may lead to unreliable information and feelings of frustration (Chen et al., 2018; and Lai et al., 2019). While data was collected on the use of other online resources, such as specific health websites and peer-reviewed journal articles, further information regarding specific sources retrieved from the Internet search engine Google by students were not collected. In general, students in our study believed a very high amount of information to exist on human nutrition, and while they thought this information to be generally easy to find, most students were only moderately confident in knowing where to find such information. These perceptions surrounding mass information availability that is easy to find, yet a shared lack of confidence in an ability to do so, may be a reflection of students’ reliance on the Internet search engine Google to acquire this information in our study.

Students who primarily relied on books or magazines, or peer-reviewed journal articles had improved odds of having self-confidence in their nutrition information-seeking abilities. However, only 99/322 (31%) of students reported the use of these resources for their personal nutrition needs. Like the Internet, literature sources such as books and magazines can vary significantly in reputability of published information, and may contain advertisements which inadvertently promote an unhealthy lifestyle (Hassan et al., 2007). As such, students should be better educated surrounding the selection of these resources to most effectively influence their personal health and nutrition needs.

Lack of confidence in knowing where or how to find information may introduce additional consequences to students’ personal health and well-being. 34/322 (10%) of first year veterinary students indicated a lack of knowledge about healthy eating as a significant barrier to changing their eating habits, and the perceived lack of knowledge significantly associated with reduced odds of perceiving their diet as healthy (Nielson et al., 2021). Though almost all first year veterinary students indicated their PHP as a primary information source for their own general health information, less than half identified their PHP as a key resource for their own nutrition information, resulting in a dichotomy in reliance on this source. The essential role of nutrition in the prevention and treatment of various diseases is well known (Lichtenstein et al., 2006). Yet, 134/322 (41%) of first year veterinary students revealed their doctor did not provide any information on healthy diets, and another 45 (14%) claimed the information they did receive to be insufficient. Previous research investigating online HISBs have identified a significant association between unmet needs of patients, including information provided by the physician, and reliance on the Internet (Tustin, 2010). Although first year veterinary students in this study indicated a general trust towards dietary recommendations from family doctors, their perceived lack of guidance from medical professionals may be hindering the opportunity physicians have to correct potential misconceptions that students could develop from using the Internet, which may influence personal health and nutrition behaviours. In a previous study by the authors, physician recommendations were considered to be a motivating factor to changing personal eating habits by nearly half of first year veterinary students (Nielson et al., 2021). Given first year veterinary students who reported the dietary information and advice received from their doctor to be sufficient were significantly more likely to be confident in knowing where to find personal nutrition information, it is important for the PHP to regularly address nutrition during appointments. Such practices can support students’ confidence in knowing where to access reliable information and ensure the dietary needs of these patients are being met. Moreover, considering younger students in our study had improved odds of having trust in family doctors recommending certain diets in comparison to older students, specifically those aged 28 or older, it is especially critical for these younger students to have already established a sufficient nutrition-related dialogue with their family doctor in an effort to maintain these trust levels throughout adulthood. Students who reported a reliance on their PHP as a primary information source for personal nutrition needs were also associated with greater odds of trusting dietary recommendations from their family doctor. Results by van Weel (2003) suggest that physicians should approach nutritional counselling with their patients using consistent, short discussions, which can be accommodated in the time constraints of regular appointments. Research investigating pet owners’ attitudes and intentions towards nutritional guidance received from veterinarians reveals a parallel to the outcomes of our study; though pet owners revealed general trust in veterinary advice, owners perceive a need for more frequent nutritional counselling from their veterinarians (Kamleh et al., 2020a). Encouraging first year veterinary students to express interest in regularly discussing their nutritional needs with their PHP would not only support them to experience the personal benefits of this credible resource, but could also serve as a professional example to these future professionals to frequently address nutritional concerns with pet owners (Walsh et al., 2002; and Kamleh et al., 2020b).

While advice from a registered dietician was ranked as highly trusted by first year veterinary students, these health professionals were reported as a primary source for personal nutrition information by less than a quarter of respondents. Information was not collected regarding the accessibility of such services to students, although many post-secondary institutions in Canada and the US offer dietician-run programmes and appointments through their student health services. The 2007 Canadian National Physician Survey reported that 52% of family physicians collaborated with dieticians or nutritionists, and 42% offered nutritional counseling within their practice (Family physicians and nutrition counseling, 2010). These statistics increased from previously conducted surveys, and at the time of that survey, over 81% of second-year family medicine students indicated they had collaborated with dietitians or nutritionists as part of their training (Family physicians and nutrition counseling, 2010). This information points towards developing accessibility to specialised nutritional services through family physicians, yet such support was not reflected in the results of our study. While 134/322 (41%) of first year veterinary students revealed their PHP as a primary information source for personal nutrition needs, and most revealed trust in family doctors recommending certain diets, the quantity and quality of information received was generally perceived to be insufficient. These results indicate that while students may generally trust and rely on nutrition and dietary information from their doctor, they may not be receiving this type of information in the capacity that they need. Further research investigating students’ accessibility to, and perceptions of, registered dieticians, as well as PHPs for personal nutrition information is needed to better understand the HISB of this population.

Information provided to students by veterinary educators may influence student learning beyond the context of veterinary medicine; over half of first year veterinary students in our study reported university lectures as a primary information source for both personal health (191/322 [59%]) and nutrition (165/322 [51%]) needs. The high demands of veterinary medical curricula are well understood, and research in other veterinary student populations have reported information overload as a significant source of stress in these individuals (McLennan & Sutton, 2005). First year veterinary students who reported university lectures as a primary nutrition information source had improved odds of believing a very high amount of nutrition information to exist. Thus, students may already feel overwhelmed by the amount of extensive health and nutrition information they are acquiring through the veterinary curriculum. As veterinary schools develop student wellness initiatives, they may want to consider providing personal health and nutrition information that complements concurrent lectures on animal health and nutrition. Similarly, such information could be incorporated into a well-being stream in the curriculum. These approaches could benefit students by reinforcing health and nutrition concepts valuable to their studies, while promoting personal applications to well-being. Another approach could consider results by Dale et al. (2011) who suggested that online communities using Web 2.0 tools (web applications that facilitate individuals to create, share, collaborate and communicate) are largely advantageous for veterinary professionals and play an important role in continuing education. As reliance on technology continues to grow, extending such online communities to support veterinary students and professionals with personal health and well-being needs could be an accessible opportunity to guide this population in retrieving credible information from an online source (Dale et al., 2011).

Information-seeking literacy skills are essential for identifying and evaluating reputable information resources. Guiding students to reputable information sources such as peer-reviewed journal articles and trusted books or magazines is one component, as is helping them assess Internet websites for accuracy and reliability. The goal is improved self-confidence in HISB. Veterinary schools should work to ensure students have the necessary skills and resources to effectively navigate health information to endorse good HISB both academically and personally. The literature suggests that poor health literacy can hinder the ability to effectively find, comprehend, and use information to manage personal health conditions (World Health Organization, 2021; Ferguson & Pawlak, 2011). Such skills are especially important for veterinary students who are responsible for managing their own health and eventually, the health of their patients. Online, in-person, and blended approaches have been shown to be equally effective for recalling information literacy skills (Anderson & May, 2010). Assisting veterinary students to develop sound HISB will not only support them in making well-informed decisions for their personal health and nutrition but encourage future client counselling habits that align with these objectives as professionals.

There are some limitations to this study. First, given the small pilot sample of the survey and limits in language translation and generalisation, the potential challenge of item development cross-linguistically, cross-culturally, and across universities sampled should be acknowledged. Results from this study provide useful insights, however standard questionnaire biases should be considered, including social desirability, acquiescence, and item order. Further, the power of the predictive models of this data may be impacted by such biases, given the potential interaction effects of questionnaire biases with result variance. Though it was stressed to students that our questionnaire was anonymous and efforts to maintain confidentiality of personal identifying data were outlined, there was a significant difference in respondents who preferred not to disclose their primary information sources for general health versus nutrition needs. Personal nutrition may be perceived as highly personal and is a potentially sensitive topic for individuals, including health professionals. Such sensitivities have the potential to result in non-response and social desirability biases. Furthermore, we did not collect information regarding students’ health status, which could influence information-seeking habits. Thus, these aspects should be considered both in interpreting our results, and in future research and initiative efforts.

Our findings present the primary personal health and nutrition information sources relied upon by first year Canadian and US veterinary students, and their held attitudes and behaviours of related resources. Results illustrate a high reliance on Internet search engines, and a general lack of guidance from medical professionals regarding students’ personal nutrition needs. This information should encourage veterinary schools to further guide their students in developing and applying sound HISB, in order to effectively support students with personal health and nutrition decisions and behaviours, and thrive as veterinary students and future health professionals.

Conflict of Interest

The authors declare no conflicts of interest.

## Disclaimer

Knowledge Summaries are a peer-reviewed article type which aims to answer a clinical question based on the best available current evidence. It does not override the responsibility of the practitioner. Informed decisions should be made by considering such factors as individual clinical expertise and judgement along with patient’s circumstances and owners’ values. Knowledge Summaries are a resource to help inform and any opinions expressed within the Knowledge Summaries are the author’s own and do not necessarily reflect the view of the RCVS Knowledge. Authors are responsible for the accuracy of the content. While the Editor and Publisher believe that all content herein are in accord with current recommendations and practice at the time of publication, they accept no legal responsibility for any errors or omissions, and make no warranty, express or implied, with respect to material contained within. For further information please refer to our Terms of Use.
